# Factors Associated With Increased Collection of Patient-Reported Outcomes Within a Large Health Care System

**DOI:** 10.1001/jamanetworkopen.2020.2764

**Published:** 2020-04-14

**Authors:** Rachel C. Sisodia, Christian Dankers, John Orav, Bernard Joseph, Peter Meyers, Patrick Wright, David St. Amand, Marcela del Carmen, Tim Ferris, Marilyn Heng, Adam Licurse, Gregg Meyer, Thomas D. Sequist

**Affiliations:** 1 Partners Healthcare, Somerville, Massachusetts; 2 Massachusetts General Hospital, Boston; 3 Harvard Medical School, Boston, Massachusetts; 4 Brigham and Women’s Hospital, Boston, Massachusetts; 5 Harvard T. H. Chan School of Public Health, Boston, Massachusetts

## Abstract

**Importance:**

The collection of patient-reported outcomes (PROs) has garnered intense interest, but dissemination of PRO programs has been limited, as have analyses of the factors associated with successful programs.

**Objective:**

To identify factors associated with improving PRO collection rates within a large health care system using a centralized PRO infrastructure.

**Design, Setting, and Participants:**

This cohort study included 205 medical and surgical clinics in the Partners Healthcare system in Massachusetts that implemented a PRO program between March 15, 2014, and December 31, 2018, using a standardized centralized infrastructure. Data were analyzed from March to April 2019.

**Exposures:**

Relevant clinical characteristics were recorded for each clinic launching a PRO program.

**Main Outcomes and Measures:**

The primary outcome was the mean PRO collection rate during each clinic’s most recent 6 months of collection prior to January 2019. Data were analyzed using a linear regression model with the 6-month PRO collection rate as the dependent variable and clinic characteristics as independent variables. Secondary analysis used a logistic regression model to assess clinical factors associated with successful clinics, defined as those that collected PROs at a rate greater than 50%.

**Results:**

Between March 2014 and December 2018, 205 Partners Healthcare clinics were available for analysis, and 4 061 205 PRO measures from 745 028 encounters were collected. Among these, 103 clinics (50.2%) collected at a rate greater than 50%. Increased collection rates were associated with more than 50% of physicians in a clinic trained on PROs (change, 19.6% [95% CI, 9.9%-29.4%]; *P* < .001), routine administrative oversight of collection rates (change, 16.0% [95% CI, 6.6%-25.5%]; *P* = .001), previous collection of PROs on paper (change, 12.5% [95% CI, 4.7%-20.3%]; *P* = .002), presence of a clinical champion (change, 11.2% [95% CI, 2.5%-20.0%]; *P* = .01) and payer incentive (change, 10.5% [95% CI, 2.0%-18.9%]; *P* = .02).

**Conclusions and Relevance:**

These findings suggest that training physicians on the use of PROs, administrative surveillance of collection rates, and the presence of a local clinical champion may be promising interventions for increasing PRO collection. Clinics that have previously collected PROs may have greater success in increasing collections. Payer incentive for collection was associated with improved collections, but not associated with successful programs.

## Key Points

**Question:**

What are the characteristics associated with improving the collection of patient-reported outcomes (PROs)?

**Findings:**

This cohort study of 205 clinics in Massachusetts that launched a PRO program found that clinician engagement, administrative engagement, and previous PRO collection experience were associated with increased collection rates. Having a clinical champion, payer incentives, and a higher collection rate during the initial month of the program were also associated with significantly increased collection rates.

**Meaning:**

These findings suggest that health care systems that seek to implement PRO programs should focus on clinician and administrative engagement to maximize the potential for programmatic success.

## Teaser

This cohort study examines factors associated with increased collection of patient reported outcomes by clinics in a large health care system.

## Introduction

In recent years, there has been increasing interest in the collection of patient-reported outcomes (PROs), which may be the best measure of delivering value in health care, demonstrating that a patient’s symptoms are improving or worsening through their interaction with the health care system. A focus on collection of PROs can address the discordance between the patient and the physician as to the gravity and severity of symptoms,^1,2,3^ and use of PROs has led to improved overall survival in the setting of metastatic cancer.^4^ For these reasons, payers and employers are interested in the collection of PROs to assess the value of care delivered.

Despite the benefits of PRO collection, the widespread implementation of routine collection has been limited owing to clinician, staff, and patient reluctance, inadequate resources to deal with positive or unexpected results, concerns for how the data will be used, and workflow and technology challenges.^5^ While some organizations have published descriptions of their PRO programs in specific disciplines, there is a paucity of information on large-scale systemwide implementations that include diverse specialties and clinical settings. Even less is known about why individual clinics may succeed or fail in the collection of PROs and what resources must be in place to ensure successful collection. The objective of this study was to prospectively assess the factors associated with success or failure of a clinic’s PRO program within a diverse, integrated health care system.

## Methods

### Study Setting, Participants, and Implementation Model

This cohort study was conducted within the Partners Healthcare system, a large, nonprofit health care system in Massachusetts. Partners Healthcare comprises 2 academic medical centers (Brigham and Women’s Hospital and Massachusetts General Hospital) as well as 7 community hospitals, 3 specialty hospitals, and a large network of community-based physician office practices. In 2014, Partners Healthcare deployed a central infrastructure to support the collection of PROs throughout the system. Collection of PROs was voluntary, and the implementation model allowed clinical specialties to choose whichever condition, PRO, and PRO measure (PROM) they deemed clinically appropriate. Standardized support included procurement of tablets, optimization of clinic wireless internet, building the PROMs in the electronic health record (EHR), assigning the PROMs to patients, and ensuring that the PROMs flowed into the enterprise data warehouse in a high-fidelity fashion. Each clinic was assigned a project specialist who provided training to the front desk staff, clinic administrators, clinic nurses, medical assistants, and physicians on how to use PROMs and find them in the EHR and best practices (if they existed) for that particular PROM. It was the responsibility of the clinic to enforce that all clinicians and administrators received training. Weekly reporting on collection rates was centrally provided to clinics, but it was the responsibility of clinic administration to act on these results should collection rates decrease.

For our analysis, clinics were included if they launched a PRO program between March 15, 2014 (program inception), and December 31, 2018. To be included in the study, clinics had to have at least 6 months of collections data. This report follows Strengthening the Reporting of Observational Studies in Epidemiology (STROBE) reporting guideline for cohort studies, and institutional review board approval and participant consent were waived per Partners Healthcare protocol for studies undertaken for the sake of quality improvement.

### Data Sources

The study population included clinics that launched PRO collection programs between March 2014 and December 2018. Clinical characteristics were recorded by project specialists in project logs at the time of clinic launch and in an ongoing manner. Data detailing number and type of completed PROMs questionnaires, as well as collection rates, were obtained from our enterprise data warehouse. Each PROM was assigned in questionnaire sets assigned for a particular indication (eg, the PROM for orthopedics knee pain included Patient-Reported Outcomes Measurement Information System [PROMIS] Global-10, PROMIS Physical function, Knee Injury and Osteoarthritis Outcome, PROMIS Anxiety, and PROMIS Depression questionnaires). No more than 1 questionnaire set was administered per any given patient encounter. For this analysis, the unit of analysis was completion rate per assigned questionnaire set.

### Outcome Measures

Our primary outcome was the mean PRO collection rate per clinic, defined as the clinic’s mean collection rate during the most recent 6 months of collection. Clinics were excluded if they did not have at least 6 months of data, were missing more than 3 months of data within the most recent 6 months, or had incomplete assessment of the included clinical characteristics. A secondary outcome was the primary outcome dichotomized at a mean PRO collection rate of 50% or greater vs less. In this way, a clinic was designated as successful if its mean collection rate in the 6 months prior to January 2019 was 50% or greater.

### Clinical Characteristics Potentially Associated With Collection Rates

To our knowledge, there are no previously published variables that are associated with the success of a PROs program. At time of program inception, 9 characteristics were believed to be relevant based on previous large-scale program implementations within Partners Healthcare and basic tenets of implementation science^6^ and included in our model. To minimize bias, variables were recorded in a prospective fashion by project specialists who were not involved in the design of this study and stored in project logs.

#### Academic Medical Center

Partners Healthcare has clinics located in academic medical centers as well as freestanding community health clinics. Clinics were stratified by whether they were located in an academic medical center vs a community center.

#### Early Adoption

Within the early years of our program, there was a learning curve on how to implement a PROs program. Clinics were labeled as early adopters if they launched a PRO program in the first 2 years of our program, from March 2014 to December 2016.

#### Payer Incentive

Partners Healthcare includes PRO collection in several specialties in performance-based contracts with payers. If a clinic’s collections were included in a payer contract with financial penalty at stake, we listed it as having a payer incentive.

#### Previous PRO Collection

Some clinics collected PROs on paper prior to our electronic platform. These clinics were categorized as having previous PRO collection.

#### Clinician Engagement

Our implementation model included standardized physician training on PROMs. The rate at which physicians participated in this training was overseen by each individual clinic. If more than 50% of clinicians in a clinic were trained, the clinic was listed as having high clinician engagement.

#### Administrative Engagement

Monthly dashboards were provided showing clinic collection rates. If the administrator of a clinic downloaded and used these reports to improve collections, the clinic was listed as having administrative engagement.

#### Leadership Mandate

Clinics were classified as having leadership mandate if the PRO program was launched at the request of the chair or senior medical executive of a department. In addition, clinics were included as having leadership mandate if departmental leadership required PROMs to be collected as part of a quality incentive for the department.

#### Clinical Champion

Clinics were considered to have a clinical champion if there was a physician, outside of the designated department leader and physically located within the clinic, who took responsibility as the clinical contact for the PRO program and was listed as the clinician responsible for performance.

#### Platform Transition

In the early years of the PRO program, PRO collection occurred electronically via a third-party vendor and was stored outside the EHR. By January 2016, the entire system had converted to a single EHR (Epic Systems) and our PRO collection platform was transitioned from the third-party vendor to the EHR solution. The EHR platform allowed for collection either in the clinic via tablet or kiosk or at home via the patient portal. During the transition, programs that had previously collected PROs using the third-party vendor might have PRO collection interrupted for months. If a clinic originally used a third-party non-EHR vendor, they were listed as undergoing platform transition.

#### Initial Collection Rate

In addition to the 9 clinic characteristics considered main variables, we considered initial collection rate for its potential association with long-term performance in the PRO program. Initial collection rate was defined as the collection rate during the first month after PRO program launch that a clinic’s collection rate exceeded 10%. We selected this threshold because low collection rates can be achieved via the patient portal alone with no engagement of the clinic. A cutoff collection rate of greater than 10% ensures clinic engagement via distribution of tablets in the waiting room.

### Statistical Analysis

Our primary analysis used a linear regression model with each clinic’s mean collection rate as the dependent variable and 10 clinic characteristics as independent variables. Since our goal was to identify modifiable risk factors that could become interventions for clinics wishing to initiate similar PRO programs, our model did not include other clinic characteristics, such as specialty. Collinearity between the risk factors was assessed using the Belsey-Kuh-Welch diagnostic. Since the highest condition index was 4.1, well below the problem threshold of 30, we believe that the model with all risk factors simultaneously present does not mask any important effects. Model residuals were also tested and passed the Shapiro-Wilk test for normality (*P* = .18)

Additionally, although incremental improvement of PRO collection is important, programs may desire to collect PROs at rates high enough to make valid inferences about the population being studied. To better understand the attributes of clinics that collected PROs at a high rate, we also ran a logistic regression model to identify clinic characteristics associated with success (ie, a collection rate of ≥50%).

All analyses were carried out using SAS statistical software version 9.4 (SAS Institute). *P* values were 2-tailed and considered statistically significant at *P* < .05. Data were analyzed from March to April 2019.

## Results

Among 231 eligible clinics, we excluded 23 clinics for missing 3 or more months of data within the most recent 6 months and 3 clinics for having incomplete recording of clinical characteristics. From the remaining 205 clinics representing 56 disciplines (eTable in the Supplement), Partners Healthcare collected 4 061 205 individual PRO questionnaires from 745 028 encounters. One questionnaire set was administered per encounter. Most collections (618 374 PROMs [83.0%]) occurred within the clinic via tablet, with the remainder (126 654 PROMs [17.0%]) collected via the patient portal. A total of 700 326 questionnaire sets (94.0%) that were started resulted in completion of all PROMs within that set.

Clinical characteristics are reported in Table 1. Most clinics (152 clinics [74.1%]) were in academic medical centers, and less than half of clinics (93 clinics [45.4%]) launched their PRO programs in the first 2 years of the program (ie, early adoption). Administrative engagement was present in 134 clinics (65.4%), 123 clinics (60.0%) had a leadership mandate to collect PROMs, and 99 clinics (48.3%) had clinician engagement (Table 1). Nearly all clinics had 3 to 6 of the clinical characteristics present (187 clinics [87.8%]), and more than half of clinics had 5 to 6 characteristics (110 clinics [53.7%]). Using a linear regression, an increase in the number of clinical characteristics was associated with a significant increase in PROMs completion rates (change per additional characteristic, 11.9% [95% CI, 9.1%-14.5%]; *P* < .001).

**Table 1.** Characteristics of Included Clinics

Characteristic	No. (%) (N = 205)
Clinic type	
Medical	152 (74.1)
Surgical	35 (17.1)
Mental and behavioral health	18 (8.8)
Duration in the PRO program, mo	
Median (range)	20 (4-44)
4-12	69 (33.6)
13-24	55 (26.8)
25-36	49 (23.9)
37-44	32 (15.6)
Clinical characteristics	
Presence in an academic medical center	151 (73.7)
Early adoption	93 (45.4)
Payer incentive	110 (53.7)
Previous patient-reported outcomes collection	118 (57.6)
Clinicians engagement	99 (48.3)
Administrative engagement	134 (65.4)
Leadership mandate	123 (60.0)
Clinical champion	64 (31.2)
Platform transition	39 (19.0)
Characteristics present, No.	
1	8 (3.9)
2	9 (4.4)
3	34 (16.6)
4	36 (17.6)
5	58 (28.3)
6	52 (25.4)
7	7 (3.4)
8	1 (0.5)
9	0

Abbreviation: PRO, patient-reported outcome.

Half of the included clinics (103 clinics [50.2%]) were considered successful clinics with a sustained collection rate of 50% or greater. The median time to achieve a collection rate of greater than 50% was 38 (95% CI, 36-40) months. Of the remaining clinics, 33 (16.1%) had collection rates less than 10%, and 69 (33.7%) had intermediate performance (ie, collection rates >10% and <50%). The highest collection rates by specialty were observed in primary care and internal medicine (112 123 PROMs [15.0%]), pediatrics (83 532 PROMs [11.2%]), orthopedics (129 069 [17.3%]), and oncology (104 344 PROMs [14.0%]) (eTable in the Supplement). There was no overrepresentation of any particular specialty in clinics that had unsuccessful PRO programs.

In multivariable analysis of PROMs collection rates, clinician engagement was associated with an increase of 19.6% (95% CI, 9.9%-29.4%; *P* < .001) in the collection rate. Administrative engagement was associated with an increase of 16.0% (95% CI, 6.6%-25.5%; *P* = .001), previous PRO collection was associated with an increase of 12.5% (95% CI, 4.7%-20.3%; *P* = .002), presence of a clinical champion was associated with an increase of 11.2% (95% CI, 2.5%-20.0%; *P* = .01), and presence of payer incentives was associated with an increase of 10.5% (95% CI, 2.0%-18.9%; *P* = .02) (Table 2). Each 10% increase in collection rate in the initial month was associated with a 2.6% (95% CI, 1.2%-4.9%; *P* < .001) increase in the final collection rate. Presence in an academic medical center, leadership mandate, early adoption, and platform transition were not associated with increased rate of collection. 

**Table 2.** Associations of Clinical Characteristics With Increased PROs Collection Rates and Successful Collection

Factor	Collection rate		Successful collection^a^	
	Change (95% CI), %	*P* value	Odds ratio (95% CI)	*P* value
Initial month rate ≥10%	2.6 (1.2 to 4.9)	<.001	1.25 (1.04 to 1.51)	.02
Academic medical center	2.0 (−6.2 to 10.1)	.63	1.09 (0.38 to 3.11)	.87
Early adopter	−5.0 (−12.9 to 2.8)	.20	1.24 (0.44 to 3.49)	.69
Payer incentive	10.5 (2.0 to 18.9)	.02	1.83 (0.63 to 5.31)	.27
Previous PROs collection	12.5 (4.7 to 20.3)	.002	5.83 (2.16 to 15.69)	<.001
Clinicians engaged	19.6 (9.9 to 29.4)	<.001	4.38 (1.50 to 12.80)	.007
Administrative engagement	16.0 (6.6 to 25.5)	.001	6.76 (2.01 to 22.76)	.002
Leadership mandate	3.8 (−6.2 to 13.8)	.45	1.75 (0.50 to 6.10)	.38
Clinical champion	11.2 (2.5 to 20.0)	.01	3.36 (1.06 to 10.61)	.04
Platform transition	4.2 (−4.8 to 13.1)	.92	2.41 (0.79 to 7.41)	.12

Abbreviation: PRO, patient-reported outcome.

^a^ Successful clinics were those with collection rates 50% or greater.

Among clinics with high collection rates, all of the same clinical characteristics, except payer incentive, remained significantly associated with implementation of a successful PROMs program (clinician engagement: odds ratio [OR], 4.38 [95% CI, 1.50-12.80]; *P* = .007; administrative engagement: OR, 6.76 [95% CI, 2.01-22.76]; *P* = .002; previous PRO collection: OR, 5.83 [95% CI, 2.16-15.69]; *P* < .001; clinical champion: OR, 3.36 [95% CI, 1.06-10.61]; *P* = .04; payer incentives: OR, 1.83 [95% CI, 0.63-5.31]; *P* = .27) (Table 2).

## Discussion

This cohort study examined the factors associated with increased PROM collection rates and PRO program success in the largest PRO program reported in the literature, to our knowledge. After collecting more than 4 million questionnaires across hundreds of clinics, we found that several factors were associated with increasing a clinic’s PRO collection rate. The strongest associations were with physician and administrative engagement, but previous collection of PROs, presence of a clinical champion, and inclusion in a payer incentive contract were also associated with increased performance. In addition, clinics that performed well in their initial month of collection also tended to perform better over time. When examining clinics based on whether they were successful (ie, ≥50% collection), all of these factors were still relevant with the exception of inclusion in a payer incentive contract.

A key takeaway from our findings is that health care systems interested in collecting PROs should focus primarily on engaging physicians in the value of the process and training them to be adept at the collection of PRO data. Additionally, administrators should not be ignored in the process, as their engagement and oversight of collection numbers was strongly associated with success.

Another key finding is that mandating PRO collection, either by local leadership or payers, was not significantly associated with the creation of a successful program. Inclusion of a clinic’s PROs in a payer contract was associated with an increase in PRO collections; however, it was not associated with clinics collecting successfully in a sustained fashion. This finding undermines the idea that payers could drive PRO collection to a meaningful level based on the implementation of PRO performance measures.^7^ Instead, our data suggest that the most important factor associated with a clinic’s success is physician engagement with the process.

An additional, pragmatic finding of our study is that improving a clinic’s collections in the first month after launch was associated with improved collection over time. An additional pragmatic finding of our study is that improving a clinics collection in the first month after launch was associated with improved collection over time. This supports the importance of ensuring that local wireless internet, assignment logic of questionnaires, and training of physicians and administrators prior to launch. This clinical observation was supported by our models. Over time, we have begun to treat the month of launch as a critical junction in the lifecycle of a clinical PRO program and to focus exhaustively on ensuring that both technical and human errors are reduced to a minimum.

Finally, although the collection of PROs is an area of intense interest, detailed reports on implementation are generally lacking and either focus on implementation steps or single disease sites, such as PROs in patients at orthopedics clinics, or questionnaires assigned rather than questionnaires completed.^5,8,9^ This study is an important contribution to the literature as it outlines some modifiable practices and characteristics that are associated with improving PRO collection. Furthermore, it demonstrates that even with costly and intensive central support, programs are less likely to succeed without physician engagement. This finding can inform the strategy of other health care systems that wish to implement PRO programs and serves as a cautionary message to systems seeking to drive the collection of PRO data via mandate or financial penalty.

### Strengths and Limitations

Our study has some strengths. One is that it is a prospective analysis of hundreds of medical and surgical clinics launching a PRO program within a standardized implementation model in an integrated delivery system. The standardized support model allowed us to make direct comparisons among attributes of the clinics, their collection rates, and whether they ultimately developed a successful program.

Our study also has some limitations. One is that it is impossible to collect all attributes that may affect an individual clinic’s collection rate. For example, clinician engagement was the factor most strongly associated with improved collection of PROs; for the purposes of this study, we defined clinician engagement as more than half of the physicians in a clinic trained on PROMs. Yet this simple concept includes a tremendous amount of nuance. The Consolidated Framework for Advancing Implementation Science^6^ would describe physician engagement as a complex concept at the nexus of outer and inner domains of a health care setting. Therefore, engagement is a reflection of how those physicians interact with external organizations, their individual patient panel, peer pressure from their own partners, competition with colleagues in other health care settings, external policies, structural characteristics of the organization, individual autonomy, and cultural attitudes about change. Defining engagement as attending a clinical training, by necessity, oversimplifies the concept. Furthermore, in a health care setting in which physicians have broad autonomy and freedom, such as Partners Healthcare, electively presenting for training inherently means a higher degree of engagement than in a health care system in which physicians are forced to undergo such a training. As such, caution must be taken when applying these results to other organizations. An additional limitation is that while the prospective collection of data minimizes the potential for bias, clinic attributes were assessed by project specialists and could be subject to some degree of bias in interpretation. Furthermore, some variables (eg, administrative surveillance) were reliant on clinic reporting.

## Conclusions

The findings of this cohort study suggest that widespread, multispecialty, and multisetting PRO collection within EHRs is feasible, but success is not guaranteed. Clinician and administrative engagement, previous PRO collection, the presence of a local clinical champion, and payer incentive were all associated with increasing collections, but only clinician and administrative engagement, previous PRO collection, and presence of a local champion were associated with clinical program success. As health care systems seek to expand PRO collection, it is critical to foster and encourage these criteria, or implementation efforts may risk failure.

## Notes

Supplement. **eTable.** Included Clinics in Partners Healthcare System Collecting Patient-Reported Outcomes

## References

[1] Laugsand EA, Sprangers MA, Bjordal K, Skorpen F, Kaasa S, Klepstad P Health care providers underestimate symptom intensities of cancer patients: a multicenter European study. Health Qual Life Outcomes. 2010;8:. doi:10.1186/1477-7525-8-104 20858248 PMC2949821

[2] Basch E, Jia X, Heller G, Adverse symptom event reporting by patients vs clinicians: relationships with clinical outcomes. J Natl Cancer Inst. 2009;101(23):1624-. doi:10.1093/jnci/djp386 19920223 PMC2786917

[3] von Eisenhart Rothe A, Bielitzer M, Meinertz T, Limbourg T, Ladwig KH, Goette A Predictors of discordance between physicians’ and patients’ appraisals of health-related quality of life in atrial fibrillation patients: findings from the Angiotensin II Antagonist in Paroxysmal Atrial Fibrillation Trial. Am Heart J. 2013;166(3):589-596. doi:10.1016/j.ahj.2013.05.020 24016511

[4] Basch E, Deal AM, Dueck AC, Overall survival results of a trial assessing patient-reported outcomes for symptom monitoring during routine cancer treatment. JAMA. 2017;318(2):197-198. doi:10.1001/jama.2017.7156 28586821 PMC5817466

[5] Basch E Patient-reported outcomes—harnessing patients’ voices to improve clinical care. N Engl J Med. 2017;376(2):105-108. doi:10.1056/NEJMp1611252 28076708

[6] Damschroder LJ, Aron DC, Keith RE, Kirsh SR, Alexander JA, Lowery JC Fostering implementation of health services research findings into practice: a consolidated framework for advancing implementation science. Implement Sci. 2009;4:50. doi:10.1186/1748-5908-4-50 19664226 PMC2736161

[7] Safran DG Feasibility and value of patient-reported outcome measures for value-based payment. Med Care. 2019;57(3):177-179. doi:10.1097/MLR.0000000000001069 30664612

[8] Biber J, Ose D, Reese J, Patient reported outcomes—experiences with implementation in a university health care setting. J Patient Rep Outcomes. 2018;2:34. doi:10.1186/s41687-018-0059-0 30175316 PMC6097980

[9] Basch E, Barbera L, Kerrigan CL, Velikova G Implementation of patient-reported outcomes in routine medical care. Am Soc Clin Oncol Educ Book. 2018;38:122-134. doi:10.1200/EDBK_200383 30231381

