# Supplementary material for: Factors Associated With Increased Collection of Patient-Reported Outcomes Within a Large Health Care System
Source: JAMA Netw Open. 2020 Apr 14;3(4):e202764. doi: 10.1001/jamanetworkopen.2020.2764 (PMC7156989; doi:10.1001/jamanetworkopen.2020.2764)
Supplement: Supplement. — eTable. Included Clinics in Partners Healthcare System Collecting Patient-Reported Outcomes [file jamanetwopen-3-e202764-s001.pdf]

Supplementary Online Content

Sisodia RC, Dankers C, Orav J, et al. Factors associated with increased collection of patient-reported outcomes within a large health care system. *JAMA Netw Open*. 2020;3(4):e202764. doi:10.1001/jamanetworkopen.2020.2764

eTable. Included Clinics in Partners Healthcare System Collecting Patient-Reported Outcomes

This supplementary material has been provided by the authors to give readers additional information about their work.

© 2020 Sisodia RC et al. *JAMA Network Open.*

eTable. Included Clinics in Partners Healthcare System Collecting Patient-Reported Outcomes

Type of Clinic	Number of Clinics	% of Total Clinics collecting PROs within the Enterprise (n=205)	Number of Questionnaire sets collected by clinic	% of Total Questionnaire sets collected within the Enterprise (n=745,028)
Medical & Behavioral Health Clinics				
Internal Medicine	32	15.6%	112,123	15.0%
Oncology	32	15.6%	104,344	14.0%
Pediatrics	15	7.3%	83,532	11·2%
Family Medicine	16	7.8%	49,994	6·7%
Psychiatry and Behavioral Medicine	16	7.8%	31,403	5·0%
Neurology	11	5.3%	28,311	3·8%
Physical Medicine & Rehabilitation	5	2.4%	26,759	3·6%
Cardiology	3	1.5%	17,955	2·4%
Adolescent Medicine	2	1%	775	<1%
Allergy/Immunology	2	1%	1,806	<1%
Dermatology	2	1%	623	<1%
Endocrinology	2	1%	47	<1%
Gastroenterology	1	0.5%	1,873	<1%
Genetics	2	1%	337	<1%
Infectious Disease	1	0.5%	314	<1%
Infusion	5	2.4%	1,959	<1%
Integrative Medicine	1	0.5%	3,947	<1%
Nephrology	2	1%	1,268	<1%

© 2020 Sisodia RC et al. *JAMA Network Open.*

Obstetrics	1	0.5%	3,181	<1%
Occupational Therapy	3	1.5%	302	<1%
Palliative Care	2	1%	384	<1%
Pediatric Endocrinology	2	1%	668	<1%
Pediatric Gastroenterology	2	1%	444	<1%
Pain Medicine	3	1.5%	5,967	<1%
Physical Therapy	5	2.4%	3,915	<1%
Psychiatry (Child and Adolescent)	1	0.5%	3,063	<1%
Rheumatology	2	1%	6,021	<1%
Sleep Medicine	1	0.5%	2,017	<1%
Surgical Clinics				
Orthopedics	18	8.7%	129,069	17.3%
Neurosurgery	6	2.9%	57,428	7·7%
Urology	2	1%	44,051	5·9%
Hand Surgery	1	0.5%	15,645	2·1%
Bariatric Surgery	1	0.5%	567	<1%
Burn Surgery	1	0.5%	206	<1%
Cardiac Surgery	1	0.5%	1,380	<1%
Pediatric Orthopedics	1	0.5%	517	<1%
Podiatry	1	0.5%	1,418	<1%
Vascular Surgery	1	0.5%	1,415	<1%

© 2020 Sisodia RC et al. *JAMA Network Open.*
